# Acute HIV Infection in Youth: Protocol for the Adolescent Trials Network 147 (ATN147) Comprehensive Adolescent Research and Engagement Studies (CARES) Study

**DOI:** 10.2196/10807

**Published:** 2019-01-16

**Authors:** Karin Nielsen-Saines, Kate Mitchell, Tara Kerin, Jasmine Fournier, Leslie Kozina, Brenda Andrews, Ruth Cortado, Robert Bolan, Risa Flynn, Mary Jane Rotheram, Sue Ellen Abdalian, Yvonne Bryson, Adolescent Medicine Trials Network (ATN) CARES Team

**Affiliations:** 1 Department of Pediatrics David Geffen School of Medicine University of California Los Angeles Los Angeles, CA United States; 2 Department of Pediatrics School of Medicine Tulane University New Orleans, LA United States; 3 Los Angeles LGBT Center Los Angeles, CA United States; 4 Department of Psychiatry and Behavioral Sciences University of California Los Angeles Los Angeles, CA United States; 5 University of California Los Angeles Los Angeles, CA United States; 6 Tulane University New Orleans, LA United States; 7 Nova Southeastern University Fort Lauderdale, FL United States; 8 University of Central Florida Orlando, FL United States; 9 University of California San Francisco San Francisco, CA United States

**Keywords:** HIV, youth, adolescents

## Abstract

**Background:**

Early treatment studies have shown that prompt treatment of HIV with combination antiretroviral therapy (cART) can limit the size of latent viral reservoirs, thereby providing clinical and public health benefits. Studies have demonstrated that adolescents have a greater capacity for immune reconstitution than adults. Nevertheless, adolescents who acquired HIV through sexual transmission have not been included in early treatment studies because of challenges in identification and adherence to cART.

**Objective:**

This study aimed to identify and promptly treat with cART youth aged 12 to 24 years in Los Angeles and New Orleans who have acute, recent, or established HIV infection, as determined by Fiebig stages 1 to 6 determined by viral RNA polymerase chain reaction, p24 antigen presence, and HIV-1 antigen Western blot. The protocol recommends treatment on the day of diagnosis when feasible. Surveillance and dedicated behavioral strategies are used to retain them in care and optimize adherence. Through serial follow-up, HIV biomarkers and response to antiretroviral therapy (ART) are assessed. The study aims to assess viral dynamics, decay and persistence of viral reservoirs over time, and correlate these data with the duration of viral suppression.

**Methods:**

A total of 72 youth (36 acutely infected and 36 treatment naïve controls) are enrolled across clinical sites using a current community-based strategy and direct referrals. Youth are prescribed ART according to the standard of care HIV-1 management guidelines and followed for a period of 2 years. Assessments are conducted at specific time points throughout these 2 years of follow-up for monitoring of adherence to ART, viral load, magnitude of HIV reservoirs, and presence of coinfections.

**Results:**

The study began enrolling youth in July 2017 across study sites in Los Angeles and New Orleans. As of September 30, 2018, a total of 37 youth were enrolled, 12 with recently acquired, 16 with established HIV infection as determined by Fiebig staging, and 9 pending determination of Fiebig status. Recruitment and enrollment are ongoing.

**Conclusions:**

We hypothesize that the size of the HIV reservoir and immune activation markers will be different across groups treated with cART, that is, those with acute or recent HIV infection and those with established infection. Adolescents treated early who are virally suppressed will have diminished HIV reservoirs than those with established infection. These youth may be potential candidates for a possible HIV vaccine and additional HIV remission intervention trials**.** Our study will inform future studies of viral remission strategies.

**International Registered Report Identifier (IRRID):**

DERR1-10.2196/10807

## Introduction

### Background

Of the roughly 40,000 people infected with HIV each year, about 25% are youth aged between 13 and 24 years, and more than half of them are not aware of their status [1,2]. Runaway and homeless youth are particularly susceptible to substance abuse, survival sex (prostitution because of extreme need), contact with the juvenile justice system, and sexually transmitted infections (STIs), putting them at a higher risk of acquiring HIV [3,4]. Specifically, African American males engaging in male-to-male contact continue to have the highest risk of HIV [1,2]. One early study found the seroprevalence of HIV in adolescents aged between 13 and 24 years to be 5.3% [5], whereas more recent estimates from the Centers for Disease Control and Prevention (CDC) have been as high as 6.1% [1].

**Figure 1:**
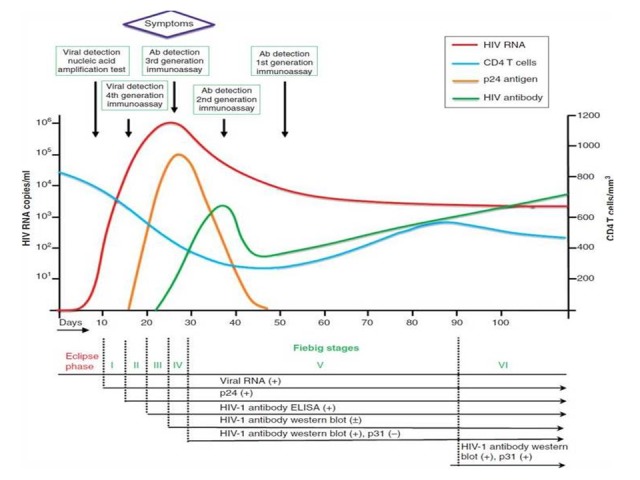
Trajectories of HIV-RNA viremia, CD4 T cells, p24 antigen and HIV antibody over the early phase of HIV infection. Sequence of appearance of different generations of HIV diagnostic assays is presented. Fiebig staging which represents a mean estimation of time from viral acquisition, divided into six phases, has also been superimposed. Eclipse phase is defined by the absence of any marker including p24 and viral RNA. Units for p24 antigen and HIV antibody are not mentioned due to the difference in magnitude. (Routy, J.P., W. Cao, and V. Mehraj, Overcoming the challenge of diagnosis of early HIV infection: a stepping stone to optimal patient management. Expert Rev Anti Infect Ther, 2015. 13(10): p. 1189-93).

It is believed that individuals who are acutely infected with HIV play a disproportionate role in the transmission of HIV [6,7]. Acute HIV infection is defined as the 4- to 5-week period [8] that occurs between initial HIV-1 exposure and development of HIV-1-specific antibodies (seroconversion). During this time, there is first a burst of viremia, which allows for the detection of p24 antigen, a core viral protein, and HIV RNA but not HIV-specific antibodies. Many patients experience a variety of nonspecific flu-like symptoms in this phase, which bear resemblance to mononucleosis-like syndrome. During this time, patients have significantly elevated HIV burden in the plasma and genital secretions, thus increasing the likelihood of transmission [9,10]. According to the commonly used Fiebig staging classification system for HIV infection, acutely infected patients do not have any detectable HIV antibodies (Fiebig stages 1-2). Recent infection is defined as the next phase in HIV infection when HIV antibodies become detectable by immunoassays but the HIV-specific Western blot (WB) can range from negative to indeterminate to incomplete (missing p31 band), which corresponds to Fiebig stages 3 to 5. This can last anywhere from 30 to 90 days postinitial infection until a full set of HIV antibodies are present [9,11]. Established infection is considered to be the time in which an immune response is fully mounted and is characterized by a plateau in HIV viremia, also known as Fiebig 6 (Figure 1). Data show that acute HIV infectivity is about 5-fold higher than established HIV infectivity [12,13].

Studies have shown that early detection and treatment of HIV infection have many clinical and public health benefits [14]. Studies of perinatally infected infants have been able to provide important insights about the pathogenesis of acute HIV infection and the need for prompt initiation of antiretroviral therapy. Molecular assays have made it possible to rapidly identify HIV in infants who have been exposed and estimate the duration of infection. On the basis of the time of detection, perinatal transmission of HIV can be classified as in-utero (during gestation), intrapartum (during labor or delivery), or via breastfeeding [15,16]. Studies in which combination antiretroviral therapy (cART) was administered to mothers intrapartum and the newborn shortly after birth showed that HIV perinatal transmission was significantly reduced by two-thirds [17,18]. HIV acquisition was also significantly antiretroviral therapy (ART) was administered to infants in the first 48 hours of life as opposed to previous standards of 3 days or older [18].

To further demonstrate the effectiveness of early antiretroviral therapy on infants, a large multicenter phase 3 trial, National Institute of Child Health and Development HIV Prevention Trials Network 040, conducted by our team of investigators revealed that cART reduced intrapartum HIV transmission by 50% [19] in high-risk HIV-exposed infants whose mothers did not receive cART during pregnancy. A few perinatally infected infants have been able to obtain a period of drug-free remission (plasma HIV undetectable following cessation of cART for more than 1 year). One example is that of a French child born in 1996 who began cART at 3 months of age with treatment for several years and, after drug interruption, experienced long-term drug-free remission that lasted over 12 years [20]. The well-described *Mississippi baby* case began ART 30 hours after birth following high-risk maternal exposure and continued treatment until 18 months of age; this infant experienced drug-free remission for 27 months [21]. Similarly, a recent report of an African child aged 9 years who was treated as an infant for a limited period around 7 weeks of age as part of the Children with HIV Early antiretroviral (CHER) clinical trial has subsequently been in HIV drug-free remission for almost 9 years [22]. These reports provide important information of potential advances in the field in infants and children, whereas little is known about adolescence.

The biggest barrier to HIV remission and cure in children and adults is the presence of latent HIV reservoirs (resting memory T cells and other sites, which contain integrated proviral DNA) [23]. These reservoirs usually reach a set point within the first 2 months of infection and serve as predictors of long-term HIV control [10,24]. When ART is discontinued, these HIV latent reservoirs allow for viral rebound to occur [25,26]. However, if cART is initiated during the acute phase of infection, it is possible to preserve the cluster of differentiation 4 (CD4) T cells and decrease the size of HIV reservoirs [24,27,19]. A period of drug-free remission may then be possible [21]. The French National Agency for Research on AIDS Visconti trial identified 14 adults that were treated during early acute infection and were able to maintain undetectable viral levels for several years after discontinuing cART [28]. Unfortunately, cART initiated after HIV has become established and is not associated with a limit in viral reservoir size or attainment of remission after cessation of cART [29,30].

Traditionally, adolescents who acquired HIV through sexual transmission have not been included in early treatment studies. Identification and adherence to ART and study visits are some of the many challenges associated with enrolling this population. However, data have shown that adolescents retain more residual thymic tissues than adults, giving them a greater capacity for immune reconstitution and CD4 T cell recovery than adults [31,32]. Therefore, it has been suggested that adolescents may be more responsive to early cART than adults with better chances of obtaining drug-free remission [24]. By identifying this population early and promptly initiating potent ART, with adequate surveillance and dedicated behavioral strategies to retain them in care and optimize ART adherence, it may be possible to significantly limit the size of their latent viral reservoirs and preserve their immune systems. This may enable them to better control HIV persistence for long term and allow them the opportunity to participate in additional strategies to induce HIV drug-free remission or become elite posttreatment controllers.

### Study Aims

This study aimed to identify and promptly initiate potent cART in acutely or recently established HIV-infected youth aged 12 to 24 years in Los Angeles and New Orleans. We hypothesize that the size of the HIV reservoir and immune activation markers will be different across groups treated with cART: those with acute or recent infection and established infection. We predict that adolescents who are treated early and are virally suppressed during acute or recent infection will have a decreased quantity of HIV reservoirs compared with those with established HIV infection. These youth with low reservoirs and preserved immune systems may be potential candidates for a possible HIV vaccine and additional HIV remission intervention trials.

We expect to recruit youth with acute and recent HIV infection from a large prospective HIV-negative high-risk youth cohort as well as from numerous community clinics. The HIV-negative high-risk youth cohorts are enrolled in a Comprehensive Adolescent Research and Engagement Studies (CARES) partner study, where youth at risk for HIV acquisition are followed periodically for a period of 2 years with point-of-care (POC) periodic HIV testing performed, including fourth generation HIV-1 assays (Alere Determine), GeneXpert qualitative HIV assays (Cepheid), plasma polymerase chain reaction (PCR) assays, and detuned HIV-1/2 enzyme immunoassay. Serial POC testing for syphilis, gonorrhea, and chlamydia are done at the same time points.

On initial identification of HIV infection, following written informed consent, youth are enrolled into the study, with confirmatory HIV RNA performed as well as a standard HIV fourth generation antigen-antibody assays. Study youth have a clinical visit with history, physical examination, and detailed medical or behavioral questionnaire performed. Youth have HIV genotypic susceptibility testing performed and basic hematologic and chemistry panels assessed. They are evaluated for concurrent infections, including syphilis; hepatitis A, B, and C; chlamydia; gonorrhoea; tuberculosis; and cytomegalovirus (CMV). Potent cART is prescribed at the enrollment visit (according to the standard of care procedures or Department of Health and Human Services [DHHS] guidelines and preferably using a single daily pill), and whole blood is collected to perform tests to determine Fiebig staging [11]. Serial follow-up blood samples are obtained in subsequent visits for HIV biomarkers and to monitor response to cART. The aim was to describe and compare viral dynamics, viral decay, and persistence of viral reservoirs in individuals who are acutely, recently, or chronically infected over time, with results correlated with duration of viral suppression. These assays used to measure these parameters include quantitative HIV RNA PCR; measurement of proviral DNA by digital droplet PCR; and studies of HIV-specific immunity, including cellular immunity, cytotoxic T cell responses, immune activation markers, and HIV neutralizing antibody.

Due to the relatively short study duration of 2 years and the need for continuous prolonged viral suppression following cART, we have not included a planned treatment interruption protocol. It is expected that some youth may stop or interrupt therapy because of unanticipated reasons. In these cases, we will strive to reimplement therapy as soon as possible and, meanwhile, will make every effort to closely monitor clinical outcomes and track disease progression. We will definitely strive to re-establish continued cART as soon as possible, particularly to avoid onset of clinical symptoms or presence of detectable plasma viremia >1000 copies/mL. For this purpose, we have a team of counselors and adherence specialists at every visit to encourage ART maintenance. The duration of ART-free HIV remission will be assessed in this subpopulation, and repeat studies of HIV viral reservoirs will be done at baseline and every 2 to 4 months during the ART-free period. Ultimately, in addition to quantifying the viral reservoirs of this unique population, we hope that our overall study will lead to the development of a prolonged HIV remission strategy.

## Methods

### Design and Population

This is a longitudinal strategic prospective treatment study that identifies, promptly treats, and follows a cohort of adolescent or young adults aged 12 to 24 years who have acute, recent, or established HIV infection. It will measure the effects of early intensive antiretroviral therapy on the establishment and persistence of HIV-1 reservoirs and HIV-1-specific immunity in acutely or recently HIV-infected youth compared with newly diagnosed youth with established infection lasting over 6 months. All youth will be treated according to the standard of care and followed for a period of 24 months.

This study is the first of its kind to enroll a highly at-risk population of HIV-infected adolescents or young adults in the United States. In 2015, among youth aged 13 to 24 years diagnosed in the United States, 81% were gay and bisexual males. Of newly diagnosed male youth, 55% were black, and 24% were Latino [33]. Among youth, an HIV seroprevalence study showed a 5.3% homelessness rate and considered homeless youth to be at highest risk of infection [5]. We have elected to target and enroll youth from 2 HIV epicenters, with large populations of infected and at-risk youth in Los Angeles and New Orleans.

In 2013, a total of 1820 Los Angeles County residents were reported as newly diagnosed with HIV infection, more than that of other urban cities, including Cook County, New York County, Miami-Dade County, and Harris County [34]. Los Angeles contains 6 geographic *hotspots*, which include the following areas: Metro, San Fernando Valley, South Los Angeles, East Los Angeles, San Gabriel, and South Bay [34]. According to a 2015 surveillance report, the New Orleans Metairie area was ranked third based on rate (per 100,000) of HIV diagnosis [33]. The Adolescent Trials Network (ATN) 110 Study in New Orleans screened a total of 200 gay, transgender, and bisexual youth between January and September 2013 and found 9 to be acutely infected, demonstrating the magnitude of the problem in this population [35].

Over a 2-year duration, ATN147 is attempting to enroll a total of 72 youth across clinical sites in Los Angeles and New Orleans. Criteria for study recruitment include (1) age of 12 to 24 years, (2) a positive HIV result (Alere rapid test and GeneXpert HIV qualitative PCR), (3) ability and willingness to provide written informed consent, and (4) willingness to initiate cART. For a physician to treat a participant, they must be willing to follow DHHS guidelines for antiretroviral naïve adolescents and adults [36], including the management of acutely HIV-infected adolescents and adults as outlined in the guidelines.

Youth can be excluded from the study according to the following criteria: (1) prior ART use (>1 week), (2) active drug or alcohol use or dependence that, in the opinion of the site investigator, would interfere with adherence to study requirements, (3) any acute, chronic, or recent and clinically significant medical condition that, in the opinion of the site investigator, would interfere with adherence to study requirements or jeopardize the safety or rights of the participant, (4) chronic or recurrent use of medications that modify host immune response, for example, oral or parenteral steroids and cancer chemotherapy, (5) clinical treatment with an ART regimen less effective than those recommended by DHHS HIV clinical guidelines, and (6) enrollment in an experimental ART regimen.

### Study Sites and Recruitment

**Figure 2:**
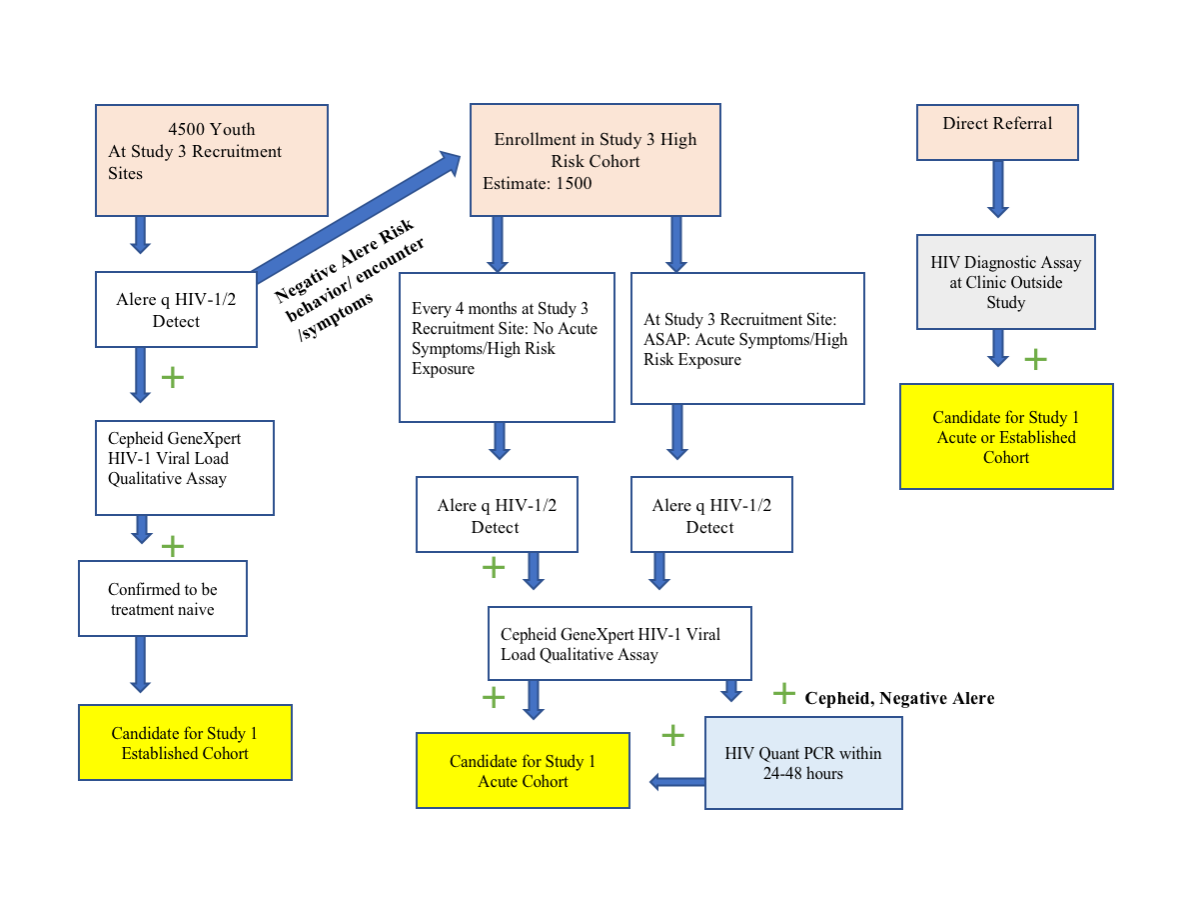
Recruitment study flow. PCR: polymerase chain reaction.

Participating ATN147 study sites include David Geffen University of California at Los Angeles (UCLA) School of Medicine, Department of Pediatrics, Division of Infectious Diseases, the Los Angeles lesbian, gay, bisexual, and transgender (LGBT) Center, and Tulane University School of Medicine, and Department of Adolescent Medicine. Identification and enrollment of acutely or recently infected adolescents are challenging. To address this challenge, we have 2 distinct methods of enrollment: ATN149 CARES and direct referrals as illustrated in Figure 2.

#### Method 1: Adolescent Trials Network 149: Cost-Efficient Interventions for Youth at Risk for HIV

ATN149 CARES is a study of high-risk HIV seronegative youth who are followed prospectively as part of a community-based strategy conducted by our group of investigators in U19 HD089886-02, a set of coordinated study that concurrently addressed youth living with HIV and seronegative youth. The study initially screens 4500 at-risk youth between the ages of 13 and 24 years at recruitment sites in Los Angeles and New Orleans for clinical and laboratory evidence of HIV using POC testing such as the Alere HIV-1/2 rapid test, which indicates the presence of HIV-1 antibody and/or antigen. In this initial screening phase, if youth are found to have a positive Alere test result and are treatment naïve, they are eligible to enroll in Protocol 147 and are, thus, referred for enrollment. Fiebig staging performed at enrollment will determine the phase of HIV infection these youth are in. Youth who happen to have a positive antigen but a negative antibody test result will undergo additional assessments to determine if they are more acutely infected and will also be referred for enrollment to Protocol 147.

Youth who are found to be seronegative and are assessed as high risk for HIV acquisition based on detailed questionnaires and STI testing are eligible for enrollment into Protocol 149. The estimated sample size of the prospective cohort is 1500 youth who will undergo qualitative and quantitative testing every 4 months for HIV and other STIs over a 2-year period. If they test positive or report symptoms of acute HIV infection, they become eligible for enrollment into the acute infection Protocol 147.

#### Method 2: Direct Referrals

Eligible youth referred to the acute infection protocol 147 from urgent care sites, emergency departments, or other clinics can also be enrolled into either the acutely or recently infected cohort or the control cohort based on confirmatory diagnostic testing as long as all eligibility requirements are met.

### Diagnosis of HIV Infection and Fiebig Staging

When potential youth are referred for enrollment screening, they may have already tested positive for HIV previously through a POC diagnostic assay. Confirmation of HIV infection is performed with a fourth generation antigen/antibody combo assay followed by a rapid molecular-based test such as the GeneXpert HIV qualitative test and/or Alere q HIV-1/2 Detect along with quantitative HIV RNA and HIV genotypic susceptibility assays. The initial testing algorithm used in the protocol is the same as the one recommended by the CDC for HIV testing, and it encompasses a POC fourth generation HIV antigen/antibody assay. Positive results are confirmed in the study with a quantitative plasma HIV detection nucleic acid assay, also as recommended by the CDC in situations where acute HIV infection is suspected. Normally, 2 HIV nucleic acid assays are not performed per CDC guidelines; however, for study purposes, a qualitative nucleic acid test is first performed in the research laboratory to rule out potentially false-positive fourth generation antigen/antibody combo assay results, particularly as the study aims for same-day treatment initiation. Results for this nucleic acid qualitative assay become available the same day as the clinic visit. An HIV quantitative plasma virus load is also requested in the first study visit; however, results of this laboratory assay take 24 to 48 hours to become available. An HIV WB is performed on stored serum specimens collected on the first clinic visit for determination of Fiebig staging. The study helps inform public health guidelines by rapidly confirming whether plasma HIV viremia is present through the qualitative HIV nucleic acid assays. In this way, patients do not need to wait a few days for virologic confirmation of infection to initiate antiretroviral treatment. This is particularly important in the case of acute infection where HIV antigen/antibody combo assays may not provide definitive results in early infection and is also helpful in situations where false-positive results may occur. Additional diagnostic testing includes CMV PCR of blood; STI testing including gonorrhoea/chlamydia PCR of urine; rapid plasma regain (RPR); Hepatitis panel, which includes hepatitis A, B, and C diagnostic testing; and tuberculosis-Gold quantiFERON testing. Clinical laboratories include a complete blood count with differential and platelets and a chemistry panel, including liver and kidney function tests.

Once enrolled, youth will eventually fall into different categories according to the Fiebig Stage Classification System based on WB results (Table 1), which characterizes the progression of HIV-1 infection from exposure to seroconversion. This staging determines acute or recent or established HIV infection status. Enrollment-visit plasma samples (which are before cART initiation) are used to determine Fiebig staging. On the basis of these stages, youth are placed into 1 of 2 cohorts, which can be further divided into groups as shown in Table 1. We expect to enroll 36 youth in each cohort over a 2-year period. Our acutely or recently infected cohort consists of Fiebig stages 1 to 5 and can be identified based on HIV-1 antibody diagnostic profile. Our control cohort consists of individuals in Fiebig stage 6 who are identified by a positive WB with a p31 band.

### Treatment

Starting at the enrollment visit, all youth sign an informed consent, are prescribed cART, and clinically treated according to standard of care HIV-1 management as defined in DHHS guidelines [9]. Genotypic drug resistance testing is performed before initiation of ART to guide the selection of the regimen. Once results of drug resistance testing are available, the treatment regimen can be modified if warranted.

Our study recommends that physicians prescribe fixed-dose combination regimens, favoring once-daily integrase inhibitor–based regimens. Use of complex ART regimens with the inclusion of protease inhibitors may trigger gastrointestinal symptoms and potential loss of adherence by our adolescent or young adult study youth, whereas efficacy of integrase inhibitor (integrase strand transfer inhibitor; INSTI)–based ARTs is successful in suppressing virus more rapidly than non-INSTI- based therapy [37]. By recommending fixed-dose combination once-daily integrase inhibitor–based regimens, we can minimize pill burden and the possibility of ART side effects. We hope that doing so will promote ART adherence and patient satisfaction in a population that is generally not amenable to medication use nor has prior experience with daily medications.

The fixed-dose combination once-daily integrase inhibitor–based regimens recommended may include the single-tablet regimen elvitegravir 150 mg/cobicistat 150 mg/emtricitabine 200 mg/tenofovir alafenamide 10 mg (EVG/COBI/FTC/TAF; Genvoya Foster City, CA: Gilead Sciences, Inc; 2016) [38] or elvitegravir 150 mg/cobicistat 150 mg/emtricitabine 200 mg/tenofovir disoproxil 300 mg (EVG/COBI/FTC/ TDF; Stribild Foster City, CA: Gilead Sciences, Inc; 2016), depending on availability or other similar regimens. EVG/COBI/FTC/TAF has been approved by the US Food and Drug Administration in November 2015 [39] and is similar to the approved single-tablet regimen with TDF but uses the TAF formulation of tenofovir, which appears to have distinct safety advantages. There have been many clinical trials that have included EVG/COBI/FTC/TDF and EVG/COBI/FTC/TAF. One phase-2 study (GS-US-292-0102) treated youth with both regimens and found that, although both groups had increased viral suppression, 88.4% (99/112) of those treated with EVG/COBI/FTC/TAF had HIV-1 RNA less than 50 copies/mL at 48 weeks by snapshot analysis compared with 88% (51/58) in those given EVG/ COBI/FTC/TDF [40].

**Table 1:** Acute, recent, and established HIV infection as per Fiebig staging.

Cohort^a^ and group		Fiebig stage	Estmated time from infection (days)	HIV-1 antibody diagnostic profile
**Acutely or recently infected (estimated N=36)**				
	1	1 or 2	0-20	Nonreactive HIV-1 antibody
	2	3 or 4	20-30	Reactive HIV-1 antibody; negative or indeterminate results on the Western blot
	3	5	30-90	Reactive HIV-1 antibody; positive Western blot without p31 band
**Control or established (estimated N=36)**				
	4	6	90+	Reactive HIV-1 antibody; positive Western blot with p31 band

^a^Adapted from: Fiebig et al study [11].

**Table 2:** Schedule of evaluations.

Evaluation	Screen	Entry	Week(s)				Month(s)				
			1	2	4	8	4	8	12	18	24
Documentation of acute HIV	X	—^a^	—	—	—	—	—	—	—	—	—
Fiebig staging	—	X	—	—	—	—	—	—	—	—	—
Financial screening	—	X	—	—	—	—	—	—	—	—	—
History	—	X	—	—	—	—	—	—	—	—	—
Physical examination	—	X	X	X	X	X	X	X	X	X	X
Adherence assessment	—	—	X	—	X	—	X	X	X	X	X
Antiretroviral therapy (ART) initiated	—	X	—	—	—	—	—	—	—	—	—
HIV-1/2 enzyme immunoassay fourth generation assay	—	X	—	—	—	—	—	—	—	—	—
HIV-1 genotypic testing	—	X	—	—	—	—	—	—	—	—	—
Urine analysis	—	X	—	—	—	—	—	—	—	—	—
Complete blood count with differential and platelets	—	X	—	X	—	X	X	X	X	X	X
Liver function tests (AST^b^, ALT^c^, GGT^d^)	—	X	—	X	—	X	X	X	X	X	X
Renal function tests (blood urea nitrogen, creatine)	—	X	—	X	—	X	X	X	X	X	X
T cell subsets	—	X	—	X	—	X	X	X	X	X	X
HIV-1 RNA PCR^e^ quantitative	—	X	—	X	—	X	X	X	X	X	X
HIV-1 Western blot	—	X	—	—	X	X	—	X	X	X	X
Hepatitis B and C panel	—	X	—	—	—	—	—	—	—	—	—
Rapid plasma regain	—	X	—	—	—	—	—	—	—	—	—
Gonorrhea/chlamydia PCR of urine, oropharynx, and rectum	—	X	—	—	—	—	—	—	—	—	—
Cytomegalovirus PCR blood	X^f^	X^f^	X^f^	X^f^	X^f^	X^f^	X^f^	X^f^	X^f^	X^f^	X^f^
Pregnancy test	—	X^g^	X^g^	X^g^	X^g^	X^g^	X^g^	X^g^	X^g^	X^g^	X^g^
Reservoir study laboratories (mL)	—	30	10	10	10	10	30	10	30	10	30

^a^Not applicable.

^b^AST: aspartate aminotransferase.

^c^ALT: alanine aminotransferase.

^d^GGT: gamma-glutamyl transferase.

^e^PCR: polymerase chain reaction.

^f^If clinically suspected.

^g^Whenever pregnancy is suspected.

Other potent antiretroviral regimens may be prescribed by the physician based on availability, patient tolerability, and preference. Changes to antiretroviral regimens should be performed when necessary according to HIV management guidelines [41]. Patients who do not tolerate the ART regimens will be prescribed an alternative ART regimen as clinically indicated. The same will occur with subjects who do not achieve viral load remission, and they may be prescribed an alternative ART regimen as clinically indicated.

Youth will take study medications and come for follow-up visits with physicians at clinic sites for up to 24 months according to the schedule of evaluations (Table 2) for assessment of viral load and HIV reservoir assays, as well as monitoring of coinfections. In addition to the enrollment visit, there will be a total of 9 follow-up visits during which samples will be collected for clinical Laboratoriess and immune reservoir studies. Of the 10 visits, 8 are scheduled during the first 12 months of ART, with 2 subsequent visits performed at 18 and 24 months.

The HIV WB is a part of immune reservoir studies and does not need to be repeated if it shows a full profile Fiebig stage 6 until 1 year. If subjects cannot do both a 2-week and 4-week follow-up visit, it is acceptable to combine the 2 visits into 1 as long as all clinical laboratory assays specified for week 4 are taken. The window periods (not shown in table) are as follows: −7 days for screen; ±7 days for week 1; ±14 days for weeks 2 and 4; ±7 days for week 8; and ±14 days for 4, 8, 12, and 24 months. In the event of virologic failure (not shown in table), HIV-1 genotypic testing will be conducted. In the event of premature study or treatment discontinuation (not shown), our team will try to collect HIV-1 genotypic testing, T cell subsets, HIV RNA quantitative assay, and 30 mL for immune reservoir studies.

### Evaluations and Activities

#### Screening Visit: Day of Diagnosis

During screening, youth are asked a series of questions as part of protocol 147 procedures. If HIV diagnosis is confirmed, a baseline interview will be conducted or if previously completed in study 3, a completed assessment interview will be performed. If youth are referred from other health care providers, the baseline assessment occurs after enrollment into the acute infection Protocol 147 (Table 2).

#### Study Entry: Day 0

Although screening visit and day 0 may occur on the same day, subjects are enrolled within 48 to 72 hours of an HIV diagnosis, and every attempt is made for treatment initiation to happen on the day of the first study visit. On this day, any necessary confirmatory diagnostic testing is performed, and those who are eligible for the study will provide written informed consent for study participation. Behavioral interventions are provided, and demographic and medical histories are collected and recorded in case report forms. A physical examination is performed, and information regarding clinical signs, symptoms, findings of acute HIV infection, and clinical treatment initiation are recorded. Patients are prescribed antiretrovirals for treatment initiation at this first visit.

If youth newly identified with HIV infection report a partner with unknown or negative HIV serostatus, we invite the study participant to bring their partner to clinic for HIV voluntary testing and counseling and also for pre-exposure prophylaxis (PrEP) initiation as part of overall HIV prevention efforts. If partners are seronegative, they are prescribed PrEP and referred to another ATN companion study (ATN149), where high-risk HIV seronegative youth are followed over time.

A number of clinical laboratory assays are performed and include the following: (1) complete blood count with differential and platelets, (2) liver function tests (aspartate aminotransferase [AST], alanine aminotransferase [ALT], and gamma-glutamyl transferase [GGT]), renal function tests (blood urea nitrogen [BUN] and creatinine), (3) CMV PCR blood if clinically suspected, (4) urine analysis, (5) gonorrhoea/chlamydia PCR of urine (6) RPR, (7) Hepatitis A, B, and C panel, (8) HIV-1 genotypic testing, (9) T cell subsets, and (10) urine pregnancy test (if female) and TB-Gold quantiFERON testing. In addition to these clinical laboratory assays, 30 mL of whole blood is collected for immune reservoir studies. Pregnancy testing is also performed if applicable.

#### Study Visits Postantiretroviral Therapy Initiation: Day 7, Week 8

A physical examination is performed, and information regarding clinical signs, symptoms, and findings of acute HIV infection or side effects from medication are recorded. Viral load testing, complete blood count, and chemistries are performed. Adherence assessment is performed, and pregnancy testing performed if applicable. In addition, 10 mL of whole blood is collected for immune reservoir studies.

#### Study Visits Postantiretroviral Therapy Initiation: Weeks 2 and 4

A physical examination is performed, and information regarding clinical signs, symptoms, and findings of acute HIV infection or side effects from medication are recorded. Adherence is assessed, and the following clinical laboratory assays are performed: (1) complete blood count with differential and platelets, (2) liver function tests (AST, ALT, and GGT) and renal function tests (BUN and creatinine), (3) HIV-1 genotypic testing (if clinically indicated or detectable viremia), (4) T cell subsets, (5) HIV-1 RNA PCR quantitative, and (6) urine pregnancy test (if suspected). In addition to these clinical laboratory assays, 10 mL of whole blood is collected for immune reservoir studies. At week 4, an HIV-1 WB is performed unless previous results showed a full profile Fiebig stage 6 (in that case, HIV-1 WB will be conducted at the 1-year mark).

#### Study Visits Postantiretroviral Therapy Initiation: Months 4, 8, 12, 18, and 24

A physical examination is performed, and information regarding clinical signs, symptoms, and findings of acute HIV infection or side effects from medication are recorded. Adherence is assessed, and the following clinical laboratory assays are performed: (1) complete blood count with differential and platelets, (2) liver function tests (AST, ALT, and GGT) and renal function tests (BUN and creatinine), (3) HIV-1 genotypic testing (if clinically indicated or detectable viremia), (4) T cell subsets, (5) HIV-1 RNA PCR quantitative, and (6) urine pregnancy test (if suspected). In addition to these clinical laboratory assays, 30 mL of whole blood is collected for immune reservoir studies.

### Immune Reservoir Studies

At enrollment, collection of 30 mL of (pretreatment) whole blood is collected and used to determine Fiebig staging through the following tests: (1) HIV-1 POC antibody test (fourth generation assay), (2) HIV-1 WB, and (3) HIV-1 quantitative RNA PCR. In addition, digital droplet PCR to measure full-length and partial HIV combination DNA transcripts are performed at enrollment and for each of the 9 subsequent follow-up visits. These tests allow the evaluation of continued reservoir suppression and sustainability of antiretroviral effect while comparing HIV viral dynamics across subject groups. The total amount of blood to be obtained during each study visit should not exceed 30 mL for youth weighing <50 kg.

The primary study endpoint is 24 months following enrollment, when the amount of cell-associated HIV-1 DNA in 5 million total peripheral blood mononuclear cells (assayed by quantitative digital drop PCR) will be compared between individuals initiating ART at different Fiebig stages: 1/2 versus 3/4 versus 5 versus established infection (Fiebig 6 control arm). We will also have HIV reservoir studies assessed at 4, 8, 12, 18, and 24 months. These data will be important to assess for studies of reservoir decay as well if there is drop out or loss to follow-up or evidence of viral rebound.

### Statistical Considerations

#### Descriptive Statistics

The results of this study will be primarily descriptive. In acute cases, the Fiebig score at the time of initiation of ART and the time to suppression of plasma viremia will be summarized over time as mean (SD). The analyses of immune biomarker assessments follow the same analysis.

#### Regression Analysis

The effects of Fiebig stage on follow-up results will be assessed in these longitudinal analyses using either numerical Fiebig stage or estimated time to initiation of ART as predictors, including stage or stage by time interaction. Drop-out is also important to assess as a binary outcome and will also be assessed with the same approach. Covariates such as STI coinfection, age, race, and behavioral assessments will be considered as potential confounders and included in the regression analysis if they confound the crude analysis. In addition, quantity of provirus over time is a longitudinal analysis. If the data are primarily described as being below detectable limits or not, longitudinal logistic regression will be conducted. If the outcome is numerical, a linear longitudinal analysis to assess the level and time trend will be conducted. Regression analysis will be conducted using the statistical package R (R version 3.0.1; The R Foundation for Statistical Computing).

### Strategies for Retention and Adherence

Study youth will be receiving adherence support through a designated case manager and adherence coach, who will already be assigned to the participant to facilitate antiretroviral use, maintenance of appointments, and facilitate overall care. All DHHS guideline recommendations for enhanced adherence will be implemented for study youth. For patients in whom adherence is identified as a major challenge, we will implement directly observed therapy. To facilitate adherence, patients will be offered free transportation to clinic and will receive continuous coaching and encouragement from a designated case manager. In addition, as this study is conducted in partnership with psychiatry and clinical psychologists, they will be available and will provide support and guidance in the management of these patients.

### Human Subjects Protection and Ethical Considerations

ATN147 has been reviewed and approved by the institutional review board (IRB) of participating institutions (UCLA IRB registration IRB #16-001819). The study focuses on prompt initiation of antiretroviral treatment on an HIV diagnosis, which is in accordance with current DHHS guidelines. The antiretrovirals that study participants initiate are first-line therapeutic options for HIV. In this way, the study implements recommended standard of care management of newly HIV-diagnosed youth. Study participants are paired with counselors and interviewers or coaches who are available for all subjects. Detailed assessments are made at each study visit of the patient’s mental status and outside activities, including the presence of other STIs and substance abuse. Participants are referred to mental health care, and resources for housing and employment are made available to them. As part of our overall HIV youth program, they are also eligible to receive Ryan White’s support for medical services. They also have access to a care coordinator and social worker to assist with their needs. Subjects are able to contact a medical provider at any time to address medical questions, including mental health issues. The study provides the background infrastructure with resources and referrals, also through the clinical sites, which are well-seasoned sites in the care of youth with HIV. Study participants are screened for depression at each visit, and if they express intent to harm themselves, they are immediately put in contact with a mental health provider for further assessment. The possibility of participants being victims of violence or participating in criminal activities is thoroughly discussed during their study interviews, with resources put in place to protect and safeguard them under this scenario. Referrals are made to local organizations such as the LGBT Center and Covenant House for support and housing needs.

## Results

The study began enrolling youth in July 2017 across study sites in Los Angeles and New Orleans. As of September 30, 2018, a total of 37 youth were enrolled, 12 with recently acquired, 16 with established HIV infection as determined by Fiebig staging, and 9 pending determination of Fiebig status. Recruitment and enrollment are ongoing.

## Discussion

### Significance of Study

Acute HIV infection in youth is a strategic treatment study aimed to identify and promptly treat recently acquired HIV with ART in youth aged 12 to 24 years who are enrolled in Los Angeles and New Orleans. This population is unique as participants are of an age in which their immune systems are more mature than that of children, yet have a greater capacity for immune reconstitution and pliability of HIV reservoirs than that of adults [24]. Our study is the first of its kind to characterize reservoirs of an adolescent population, which is generally not amenable to routine clinic visits but is at a high risk of transmission. However, through the use of a multidisciplinary approach taken in collaboration with a group of behavioral scientists, we intend to follow this cohort for 2 years and have all youth enrolled by 2019 to allow for a 2-year follow-up.

### Principal Aims

 In addition to lowering transmission in this population, our goal is to uncover new data that will inform future remission studies. The inability of cART to eradicate infected cells [42] and the fact that plasma viremia rebounds quickly after treatment discontinuation [43] are reasons why remission as a functional cure is considered a more viable goal [28]. So far, studies have shown that HIV remission is possible in both perinatally infected infants and acutely infected adults [18,28]. We believe that this is definitely possible for acutely infected adolescents as well. We hypothesize that the size of the HIV reservoir and immune activation markers will be different across groups treated with cART, that is, those with an acute or recent HIV infection and those with an established infection. Adolescents treated early who are virally suppressed will have diminished HIV reservoirs than those with established infection. By quantifying viral reservoirs of this unique population, we hope to uncover new data that will be applicable to the general HIV-infected population beyond adolescents and possibly lead to the development of a prolonged HIV remission strategy in the future.

## Abbreviations

ALT: alanine aminotransferase
ART: antiretroviral therapy
AST: aspartate aminotransferase
ATN: Adolescent Trials Network
BUN: blood urea nitrogen
CARES: Comprehensive Adolescent Research and Engagement Studies
cART: combination antiretroviral therapy
CD4: cluster of differentiation 4
CDC: Centers for Disease Control and Prevention
CMV: cytomegalovirus
COBI: cobicistat
DHHS: Department of Health and Human Services
EVG: elvitegravir
FTC: emtricitabine
GGT: gamma-glutamyl transferase
INSTI: integrase strand transfer inhibitor
IRB: institutional review board
LGBT: lesbian, gay, bisexual, and transgender
PCR: polymerase chain reaction
POC: point-of-care
PrEP: pre-exposure prophylaxis
STI: sexually transmitted infection
TAF: tenofovir alafenamide
TDF: tenofovir disoproxil
UCLA: University of California at Los Angeles
WB: Western blot
